# The Role of Ophthalmic Artery Doppler in Predicting Preeclampsia: A Review of the Literature

**DOI:** 10.3390/medicina62010186

**Published:** 2026-01-16

**Authors:** Nicoleta Gana, Ancuța Năstac, Livia Mihaela Apostol, Iulia Huluță, Corina Gica, Gheorghe Peltecu, Nicolae Gica

**Affiliations:** 1 Faculty of Medicine, Carol Davila University of Medicine and Pharmacy, 020021 Bucharest, Romania; nicoleta.gana@drd.umfcd.ro (N.G.); ancuta-iuliana.nastac0720@stud.umfcd.ro (A.N.); livia-mihaela.cosma@rez.umfcd.ro (L.M.A.); iuliahuluta16@gmail.com (I.H.); mat.corina@gmail.com (C.G.); gheorghe.peltecu@gmail.com (G.P.); 2 Clinical Hospital of Obstetrics and Gynaecology Filantropia, 011171 Bucharest, Romania

**Keywords:** PE, ophthalmic artery Doppler, prediction, preterm PE, maternal-fetal outcomes

## Abstract

*Background and Objectives:* Preeclampsia (PE) complicates 2–8% of pregnancies globally, with a higher incidence in developing countries. This condition poses significant risks to maternal and fetal health, contributing substantially to maternal and perinatal mortality, particularly in cases of early-onset PE, which is associated with severe complications. This review aims to synthesize current evidence regarding the predictive utility of ophthalmic artery Doppler for preeclampsia. Current strategies focus on early prediction and prevention to mitigate adverse outcomes and reduce the economic burden of hypertensive disorders in pregnancy. The International Federation of Gynecology and Obstetrics (FIGO) recommends first-trimester screening combining maternal risk factors, mean arterial pressure, serum placental growth factor (PlGF), and uterine artery pulsatility index (UtA-PI). High-risk women are advised to take low-dose aspirin (150 mg daily) until 36 weeks of gestation. *Materials and Methods:* This review explores an innovative predictive tool for PE: ophthalmic artery (OA) Doppler. *Results:* As a non-invasive and easily accessible method, OA Doppler provides valuable insights into intracranial vascular resistance, offering potential advantages in early risk assessment, particularly for preterm PE, the most severe form of the disease. *Conclusions:* Our findings suggest that OA Doppler may serve as a promising adjunct in PE screening, enhancing the early identification of high-risk pregnancies and improving clinical outcomes. Further research is warranted to validate its role in routine prenatal care.

## 1. Introduction

Preeclampsia (PE) continues to affect 2–8% of pregnancies worldwide, with a notably higher incidence in developing countries [1,2]. It is defined as a multisystem progressive disorder characterized by the new onset of hypertension (systolic blood pressure above 140 mmHg or diastolic blood pressure above 90 mmHg) after 20 weeks of gestation. While traditionally tied to proteinuria, contemporary diagnostics recognize PE in the absence of proteinuria if accompanied by evidence of end-organ dysfunction, such as renal insufficiency, hepatic injury, thrombocytopenia, pulmonary edema, or uteroplacental compromise. This condition significantly impacts not only maternal health but also fetal and neonatal outcomes, representing a major cause of both fetal and maternal morbidity and mortality [3,4].

Early-onset PE, in particular, is associated with a markedly increased risk of severe complications such as eclampsia, placental abruption, and HELLP syndrome. Eclampsia is the most severe manifestation of this spectrum, marked by the new onset of generalized tonic–clonic seizures or unexplained coma in a patient with preeclampsia, provided the symptoms are not attributable to pre-existing neurological conditions. The clinical distinction lies primarily in the presence of central nervous system hyperexcitability; while “preeclampsia with severe features” may present with persistent headaches or visual scotomata, the transition to eclampsia represents a critical obstetric emergency requiring immediate stabilization with magnesium sulfate and expedited delivery [2].

Long-term consequences of preeclampsia include maternal cardiovascular disease, as well as preterm birth and its associated neonatal complications (small for gestational age due to intrauterine growth restriction, cerebral palsy, and chronic conditions such as type 2 diabetes, chronic hypertension, and obesity) [4].

Moreover, hypertensive disorders in pregnancy impose a substantial economic burden due to increased rates of hospitalization and medical interventions [5]. As a result, considerable efforts are being directed toward improving the prediction, prevention, and early management of PE to reduce clinical complications and economic costs.

To this end, the International Federation of Gynecology and Obstetrics recommends universal first-trimester screening based on a combination of maternal risk factors, such as mean arterial pressure (MAP), serum placental growth factor (PlGF), and uterine artery pulsatility index (UtA-PI) [4]. When used together, these parameters can predict up to 90% of preterm PE and 75% of term PE, with a false-positive rate (FPR) of 10% [6]. Women identified as high-risk should begin to take a daily dose of aspirin (150 mg) until 36 weeks of gestation, which has been shown to reduce the incidence of preterm PE by 62% [7].

In the second trimester, the use of UtA-PI alone can predict 85% of cases of preterm PE and 48% of term cases [8]. Building upon this, Nicolaides et al. proposed a second-trimester model (19–24 weeks) incorporating maternal characteristics, MAP, UtA-PI, PlGF, and soluble FMS-like Tyrosine Kinase-1 (sFlt-1), which identified over 95% of women at high risk for early PE [9]. Similarly, in the third trimester (30–34 weeks), the same model detected 90% of women who would deliver, due to PE, within the following 4 weeks [10].

**Figure 1:**
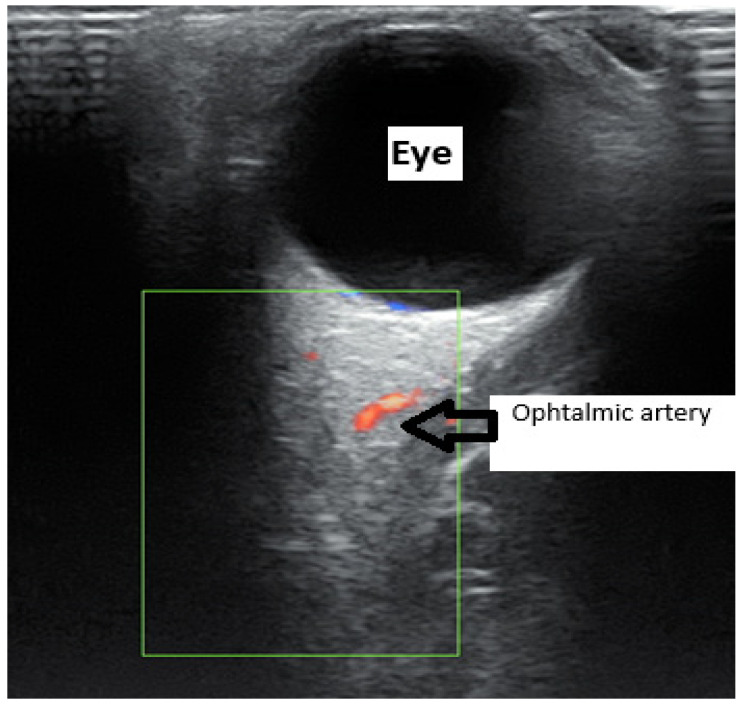
Echographic identification of the Ophthalmic artery.

**Figure 2:**
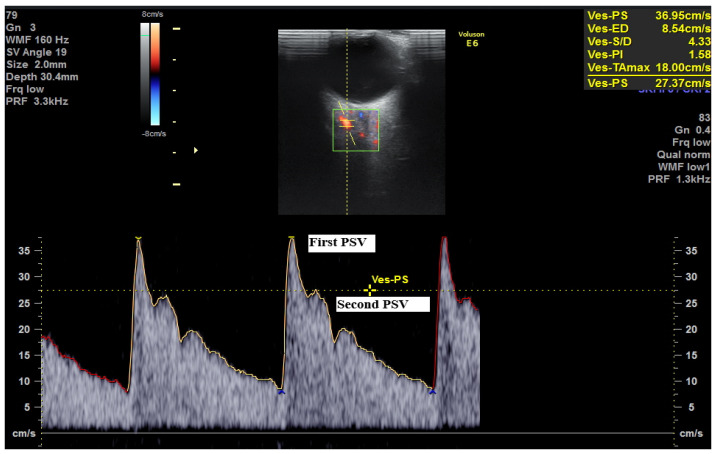
Ophthalmic artery Doppler.

In this review, we explore an additional promising tool in predicting PE: ophthalmic artery (OA) Doppler ultrasound. Several studies have highlighted the value of this method [11]. It provides a simple, non-invasive way to evaluate cerebral circulation, which is often challenging to assess directly. The technique is inexpensive, well-tolerated, easily reproducible, and requires only a few minutes [12]. Using a linear transducer (7–15 MHz), the examination is performed with the patient in a supine position, eyes closed. The probe is gently placed on the eyelid to avoid excessive pressure. The optic nerve is first identified, and with color Doppler, the OA appears medial to the optic nerve, oriented toward the inner portion of the eye (Figure 1). The Doppler gate is positioned on the vessel, capturing 3 to 5 waveforms. The insonation angle should be below 20°, and the pulse repetition frequency (PRF) is set to 125 kHz (Figure 2) [13].

The Doppler waveform of the OA is characterized by two systolic peaks. The first represents direct cardiac output transmission, while the second results from wave reflection and subsequent propagation [14]. Various Doppler indices derived from the OA have been investigated to better understand the cerebrovascular changes associated with PE [15].

## 2. Materials and Methods

To ensure transparency and a comprehensive identification of the literature, this narrative review followed a structured and rigorous search strategy. A systematic search was conducted in the PubMed/MEDLINE database from inception through January 2025, utilizing a combination of Medical Subject Headings (MeSH) and free-text keywords: (“Pre-eclampsia” OR “PE”) AND (“Ophthalmic Artery”) AND (“Doppler” OR “Ultrasonography”) AND (“Prediction” OR “Screening”). This search was designed to capture the full breadth of evidence regarding the predictive value of ophthalmic artery Doppler in preeclampsia. Study eligibility was strictly defined, targeting original, peer-reviewed, full-text primary research articles in English involving pregnant individuals where specific hemodynamic parameters—such as Peak Systolic Velocity (PSV), Resistance Index (RI), and Pulsatility Index (PI)—were evaluated. To maintain the quality of the evidence, animal studies, case reports, conference abstracts, and previous reviews were excluded. The selection process involved a standardized two-stage approach: an initial screening of titles and abstracts followed by a detailed full-text appraisal. This process was conducted independently by two reviewers to minimize selection bias, with any discrepancies resolved through consensus or consultation with a third senior reviewer. While this methodology ensures high-quality data retrieval, the primary limits of coverage include the exclusion of non-English publications and the focus on a single major database, which may omit some localized or gray literature findings.

## 3. Results

To provide a structured synthesis of the evidence, this review categorizes the findings into three primary clinical contexts, as the objectives and performance metrics of OA Doppler differ significantly across these use cases. First, we examine early screening in the first trimester, where the goal is to identify patients at risk for preterm PE to initiate preventive therapies like aspirin. Second, we evaluate later prediction and disease differentiation in the second and third trimesters, focusing on the ability of OA Doppler to distinguish PE from chronic hypertension and other gestational hypertensive disorders. Finally, we address prognostic assessment, where OA Doppler indices, particularly the peak systolic velocity ratio, are used to gauge disease severity and the likelihood of imminent delivery within one to three weeks. This organization ensures that the interpretability of performance findings is aligned with the specific clinical needs of each gestational stage.

### 3.1. Foundational Hemodynamic Evidence and Cerebral Vasodilation

The utility of the maternal ophthalmic artery (OA) as a non-invasive proxy for cerebral microcirculation was established early by Hata et al. [16]. In their 1992 report, which analyzed a small cohort of normotensive and preeclamptic (PE) patients, they hypothesized that OA flow-velocity could serve as a marker for the vascular strictures characteristic of the disease. Their results identified a decreased pulsatility index (PI) and elevated flow velocities, suggesting a paradoxical state of reduced vascular resistance in the OA of women with PE [16]. This hypothesis was further strengthened in 1997 through comparisons between normotensive pregnancies and hypertensive disorders, where significantly lower PI values in severe PE cases supported the theory of cerebral vasodilation [17]. This foundational concept was validated in larger, geographically diverse cohorts, such as a 2012 case–control study in Kano, Nigeria, which compared 96 PE patients to 96 controls. That study confirmed significantly elevated hemodynamic parameters—specifically peak systolic, end-diastolic, and peak mesodiastolic velocities—confirming profound alterations in cerebral blood flow dynamics in the PE group [18].

### 3.2. Early Screening for Preterm PE (First-Trimester Assessment)

In terms of early prediction, prospective data from 487 singleton pregnancies suggested that OA Doppler performance in the first trimester is comparable to that of the uterine artery (UtA). The incremental value of OA Doppler becomes evident when integrated into multi-modal models: combining OA data with maternal factors and Mean Arterial Pressure (MAP) identified 78% of early-onset PE cases at a 10% false-positive rate (FPR) [19].

However, the literature presents significant heterogeneity regarding the Resistance Index (RI). While some research from 2012 found no significant correlation between OA RI at 24–28 weeks and the development of PE, similar to findings by Aquino et al. in 2014 [19,20,21], these results contrast sharply with data reported by Porto et al. In the latter study, impaired placental perfusion was associated with notable differences in RI, PI, and the peak ratio (PR), with the PR emerging as the strongest independent predictor of severe PE [22].

Clinical Interpretation of OA RI: The RI is a critical Doppler parameter quantifying resistance to blood flow within the orbital circulation. It is calculated as RI = (PSV − EDV)/PSV. While normotensive pregnancies typically maintain an RI between 0.60 and 0.70, PE is characterized by significantly lower values. This shift reflects a breakdown in systemic endothelial dysfunction and compromised myogenic autoregulation, with progressive RI reductions serving as biomarkers for the transition from preeclampsia to eclampsia [20,21].

### 3.3. Later Prediction and Differential Diagnosis (Second and Third Trimester)

The clinical utility of OA Doppler extends to its ability to differentiate disease phenotypes. A longitudinal study of high-risk pregnancies demonstrated that lower RI values in the second trimester could distinguish PE from chronic hypertension, as the latter maintains RI values similar to normotensive pregnancies [23].

Further critical evidence of disease progression was found in a 2024 Indian study, where RI and PI were noticeably lower in cases versus controls. This reduction was most pronounced in eclampsia cases (velocities of 20.43 cm/s compared to 28.82 cm/s in preeclampsia), suggesting that OA indices can track the severity of hemodynamic compromise [24]. When compared to biochemical markers like the sFlt-1/PlGF ratio for predicting imminent delivery (within one week), the OA PSV-ratio showed significantly higher elevations in “imminent” cases, although multivariate analysis suggests that neither marker alone provides definitive clinical discrimination [25].

### 3.4. Conflicting Findings in Biomarker Selection

Despite the promise of OA Doppler, there is a lack of consensus on which specific index is most discriminative. A Turkish study identified the second PSV ≤ 43.75 cm/s) as the most effective parameter for PE detection [26], whereas other auxiliary analyses of 347 women found that only the peak mesodiastolic velocity (PMDV) showed statistical correlation in the second trimester [27].

Furthermore, the detection rates in screening models vary across studies. While some first-trimester models achieved a 68% detection rate by combining UtA-PI and first peak end-diastolic flow [28], a 2016 observational study reported a lower sensitivity of 45% (at 10% FPR) when combining maternal characteristics, MAP, and UtA-PI with OA Doppler in the second trimester [29]. These conflicting findings highlight the impact of gestational age and population differences on Doppler sensitivity.

### 3.5. Large-Scale Validations and Multi-Parametric Models

To address the limitations of small cohort studies, recent large-scale research has provided more robust evidence. A study of 2287 pregnancies at 35–37 weeks found that adding the PSV2/PSV1 ratio to screening models improved the detection rate for PE from 25% to 50% [30]. Follow-up studies by the same group confirmed that a comprehensive model (MAP, UtA-PI, PlGF, sFlt-1, and OA Doppler) increased the detection of PE occurring within three weeks to 88.6% [31].

This trend is corroborated by Sapantzoglou and Nicolaides, whose study of 2853 pregnancies found that the PSV ratio improved the detection rate of preterm PE from 84.9% to 89.8% [32]. The authors concluded that the PSV2/PSV1 ratio is a physiological reflection of increased afterload and ventricular wall thickness in preeclamptic women [33]. Additionally, the integration of the “Gestosis Score” with the OA Doppler PSV ratio has been shown to enhance the detection rate of preterm PE from 90% to 100%, highlighting its significant additive value to established clinical parameters [34,35].

### 3.6. Prognostic Assessment and the “Imminent Delivery” Paradigm

In the third trimester, the “incremental value” of OA Doppler is more nuanced. While some studies suggest the OA PSV ratio does not significantly enhance the predictive performance for maternal or neonatal complications already captured by the sFlt-1/PlGF ratio [36], other research identifies significant differences in PSV1, PSV2, and PI between those who develop PE and those who do not [37]. Crucially, a cohort study of 2338 pregnancies concluded that the OA PSV ratio, combined with maternal factors and MAP, could serve as an effective, lower-cost alternative to the sFlt-1/PlGF ratio for identifying imminent PE [38].

Recent data from over 4000 women reinforce that incorporating the PSV ratio improves preterm PE detection, although it offers less improvement for term PE [39]. To refine these predictions, advanced Bayesian survival models have been developed. In a study of 946 participants, a model including OA Doppler PR achieved a 100% detection rate for early-onset PE at a 10% FPR [40]. Similarly, a competing-risks model evaluating 6746 pregnancies found that the OA PSV ratio outperformed isolated biochemical markers (low PlGF or high sFlt-1/PlGF) for predicting delivery within three weeks [41]. Finally, large-scale cardiovascular assessments of 5214 pregnancies confirm that an increased PSV ratio is a consistent biomarker of peripheral vascular resistance alterations in hypertensive disorders [42].

### 3.7. Pharmacological Influence and Literature Gaps

A final critical consideration is the impact of treatment on these indices. While isosorbide dinitrate has been shown to reduce the PSV ratio and end-diastolic velocity in preeclamptic patients, magnesium sulfate appears to have no effect. A significant gap remains in the literature, as the effects of common antihypertensive agents like nifedipine, labetalol, or methyldopa on OA Doppler indices have not yet been established [43].

Table 1 summarizes the studies discussed.

**Figure 3:**
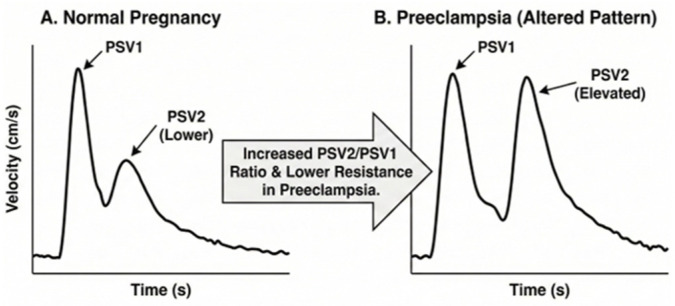
Comparative Ophthalmic artery Doppler.

To facilitate the clinical application of these findings, it is essential to distinguish between physiological and pathological ophthalmic artery Doppler patterns. Figure 3 illustrates the morphological shift observed in preeclampsia: while a normal waveform features a dominant first systolic peak (PSV1), the preeclamptic pattern is characterized by a significantly elevated second systolic peak (PSV2). This visual transition reflects a loss of normal maternal cerebrovascular autoregulation and a shift toward cerebral hyperperfusion.

The clinical reference values derived from this review are synthesized in Table 2. The thresholds listed below represent suggestive reference points based on current evidence maturity. These values should be viewed as preliminary and must be integrated with other clinical findings, as their applicability may vary depending on the specific patient context and population characteristics. Unlike normotensive pregnancies, which maintain stable vascular resistance, preeclampsia is marked by a significant reduction in the Resistance Index (RI) and Pulsatility Index (PI) alongside an elevated PSV ratio (Table 3).

## 4. Discussion

From a pathophysiological and metabolic perspective, the relationship between systemic arterial hypertension and intraocular pressure (IOP) is fundamentally governed by ocular perfusion pressure (OPP). In a healthy metabolic state, the eye utilizes myogenic and metabolic autoregulation to maintain stable blood flow despite fluctuations in MAP. However, in preeclampsia (PE), this mechanism is often compromised by systemic endothelial dysfunction and oxidative stress [46].

Metabolically, the hypertensive state triggers a systemic inflammatory response that increases the release of potent vasoconstrictors like endothelin-1 and reduces the bioavailability of nitric oxide. This leads to increased vascular resistance and altered hemodynamics in the ophthalmic artery. This systemic hyper-resistance is reflected in the eye as an increase in episcleral venous pressure, which may impede aqueous humor outflow and subsequently elevate IOP [47]. Furthermore, the high hydrostatic pressure associated with acute hypertensive spikes can disrupt the blood-aqueous barrier, potentially altering the metabolic homeostasis of the ocular environment.

Clinically, because the ophthalmic artery is a primary branch of the internal carotid artery, these changes have been proposed as a potential hemodynamic proxy for the cerebral microcirculation. Consequently, the correlation between systemic hypertension and altered ophthalmic Doppler indices suggests a state of generalized vascular maladaptation, which may offer clinical insights into the severity of maternal end-organ involvement in PE, though further validation is required to define its precise prognostic role [11,46,47].

The OA has been the focus of increasing research interest, not only for its potential role in predicting PE but also for its ability to reflect the adaptive maternal cardiovascular mechanisms associated with the condition. The current model of PE pathogenesis places the placenta at the center of the disorder [48,49]. It is well established that PE is characterized by systemic vascular dysfunction driven by an imbalance of angiogenic factors (e.g., sFlt-1, Soluble Endoglin, PlGF, and VEGF) [50].

The hypothesis that OA Doppler parameters may reflect intracerebral vascular changes has been explored in several studies (Table 1), though many are limited by small sample sizes. Early reports, such as Hata et al. (1992), highlighted the complexity of this relationship, finding a paradoxically low pulsatility index (PI) in affected women despite suspected peripheral constriction [16]. Interpretation remains variable due to the heterogeneity of indices used across studies. Recent proposals by Debs Diniz et al. (2019) to rectify waveform interpretation—noting that increased vascular resistance is often marked by decreased resistance in the diastolic component—underscore the need for specialized training and standardized measurement protocols [51].

Large-scale studies have evaluated the utility of OA Doppler across all trimesters, generally showing that the Ophthalmic Artery Peak Systolic Velocity (PSV) ratio provides modest incremental value when added to existing screening models:

- First Trimester: Adding the PSV ratio to maternal risk factors improved the detection rate of preterm PE from 46.3% to 58.4% (at a 10% FPR). However, when combined with a full suite of markers (MAP, UtA-PI, and PlGF), the improvement was more marginal (74.6% to 76.7%), and no significant benefit was observed for term PE [39].
- Second Trimester: Similar trends were noted, with the PSV ratio enhancing the detection of preterm PE from 84.9% to 89.8% when added to a comprehensive multi-marker model [32].
- Third Trimester: The inclusion of OA Doppler provided a slight increase in detection for imminent PE (onset within three weeks), moving from 84.8% to 88.6% [30,31].

These data suggest that while OA Doppler is a promising complementary tool, its additive value is most pronounced in predicting preterm rather than term PE. Furthermore, evidence suggests these indices primarily reflect maternal rather than fetal pathophysiology. A prospective study of 110 women found that while OA indices correlated with maternal disease severity, they showed no significant association with neonatal ICU admission, birth weight, or composite neonatal morbidity [52].

The clinical interpretation of these findings must account for significant study heterogeneity and common sources of bias inherent in Doppler prediction research. A primary source of variability is the population spectrum, as the predictive performance of OA Doppler fluctuates significantly depending on whether the cohort consists of low-risk screening individuals or high-risk symptomatic patients. Furthermore, acquisition protocol variability introduces technical bias; factors such as maternal supine positioning, the precise placement of the 7–15 MHz linear transducer on the eyelid, and the strict maintenance of an insonation angle below 20 degrees are essential for reproducibility but can vary significantly across different clinical settings. This variability is compounded by a lack of standardized thresholding for key parameters like the PSV ratio. Finally, the current evidence is limited by a lack of rigorous external validation in independent, large-scale multicenter cohorts, which is a necessary step to ensure the generalizability of these diagnostic markers before their formal integration into routine prenatal care guidelines.

## 5. Conclusions

The Ophthalmic Artery (OA) Doppler has emerged as a transformative tool in predictive obstetrics, offering a unique, non-invasive window into the maternal cerebrovascular adaptations that precede the clinical onset of preterm preeclampsia. By reflecting central hemodynamic shifts rather than just systemic biomarkers, OA Doppler parameters provide superior sensitivity for identifying the high-risk preterm phenotype most often associated with maternal organ dysfunction and neonatal morbidity.

The clinical utility of this technique is significantly amplified when integrated into multi-modal screening algorithms alongside traditional biochemical and clinical factors. However, for the OA Doppler to transition from a potent research tool into routine practice, the field must prioritize the standardization of acquisition protocols to ensure reproducibility, alongside the execution of large-scale, multicenter validation trials. By refining these parameters and determining their optimal weighting within existing first-trimester models, clinicians can move toward a more personalized, preventative approach that substantially reduces global maternal and fetal mortality.

## Figures and Tables

**Table 1:** Summary of the 2023 prospective study [25].

Diagnostic Parameter	Delivery Within 1 Week (Imminent)	Delivery Later (Non-Imminent)	Relative Difference and Significance
OA PSV-ratio	Markedly Elevated	Lower (Baseline)	Significantly Higher
sFlt-1/PlGF ratio	Slightly Elevated	Lower (Baseline)	Marginally Elevated

**Table 2:** Summary of the studies on ophthalmic artery Doppler in pregnancy.

Authors	Number of Cases	Gestational Age	Results
Hata et al. [16]	27	<16 weeks	PI was lower in PE.
Hata et al. [17]	76	7–40 weeks	PI was lower in PE.
Kumari et al. [18]	96	20–40 weeks	The PE group demonstrated significantly elevated hemodynamic parameters compared to controls.
Alves et al. [19]	487	1–13 weeks	A combination of maternal factors + MAP + uterine artery Doppler or OA Doppler can detect 78% of early-onset PE with 10% FPR.
Brandão et al. [20]	74	24–28 weeks	RI is not a predictive value for PE.
Aquino et al. [21]	73	24–28 weeks	RI is not a predictive value for PE.
de Oliveira et al. [22]	379	20–40 weeks	Low PI and RI and a high PR are associated with PE.
Porto et al. [23]	62	16 + 0 and 19 + 6 weeks, 24 + 0 and 27 + 6 weeks and from hospital admission to delivery	Lower RI was detected in the PE group.
Muthyal et al. [24]	70	>20 weeks	Progressive RI and PI reductions might serve as potential biomarkers for disease progression from preeclampsia to eclampsia.
Lau et al. [25]	1247	11–24 weeks	OA PSV ratio or sFlt-1/PlGF ratio alone does not provide sufficient discrimination for clinical decision-making in this PE population.
Kaplan et al. [26]	92	-	Second PSV ≥ 43.75 cm/s was demonstrated to be the most discriminative parameter for PE detection.
Matias et al. [27]	347	20–28 weeks	High PMDV and PR were detected in women with PE.
Gurgel Alves et al. [28]	440	11–14 weeks	High OA first diastolic peak was described as a marker for PE.
Praciano de Souza et al. [29]	415	18–23 weeks	OA Doppler did not promote a significant increase in the PE detection rate.
Ozdemir et al. [44]	100	>20 weeks	RI was the strongest predictor of maternal outcomes.
Matias et al. [45]	305	20–28 weeks	PSV2 is an independent predictor of PE.
Sarno et al. [30]	2287	35–37 weeks	PSV ratio can predict PE, especially within 3 weeks after assessment.
Sarno et al. [31]	2287	35–37 weeks	PSV ratio + maternal factors + MAP + PlGF increased the detection rate from 84.8% to 88.6%, at a FPR of 10%.
Sapantzoglou et al. [32]	2853	19–23 weeks	PSV ratio alone and in combination with other biomarkers is potentially useful for the prediction of PE, especially preterm PE.
Gibbone et al. [33]	2853	19–23 weeks	The increase in PSV ratio in women who develop PE is associated with increased afterload and an increase in left ventricular thickness.
Gyokova et al. [34]	200	19–23 weeks	If it is combined with established biomarkers, the Doppler index improves discriminatory power for PE risk stratification.
Shah et al. [35]	440	19–24 weeks	OA Doppler has an additive value in PE risk stratification, particularly when combined with the Gestosis Score.
Reddy et al. [36]	126	33 weeks	The addition of cardiovascular or fetal indices to the model is unlikely to improve the prognostic performance of the sFlt-1/PlGF ratio.
Saleh et al. [37]	795	28–32 weeks	High PSV and PI were statistically different in women with PE.
Lau et al. [38]	2338	35–37 weeks	PSV ratio in combination with maternal risk factors and blood pressure could potentially replace measurement of PlGF and sFlt-1/PlGF ratio in the prediction of imminent PE.
Gana et al. [39]	4066	11–13 weeks	PSV ratio was significantly increased in PE pregnancies.
Kusuma et al. [40]	946	11–13 weeks	PSV ratio in combination with other biomarkers could improve the detection rate of preterm PE.
Mansukhani [41]	6746	35–37 weeks	For PE delivery within 3 weeks, it had an 85.0% detection rate, outperforming isolated biochemical markers.
Anzoategui et al. [42]	5214	19–23 weeks	An increased PSV ratio was detected in hypertensive pregnancies.

**Table 3:** Comparative Reference Values and Diagnostic Patterns of Ophthalmic Artery Doppler Parameters in Normotensive vs. Preeclamptic Pregnancies.

Doppler Parameter	Normotensive Pregnancy (Typical Reference Range)	Preeclampsia (Typical Pattern/Proposed Cut-Off)	Clinical Interpretation of Altered Pattern
Resistance Index (RI)	0.60–0.70	Reduced (Typically <0.60; some studies suggest <0.58 for high risk)	Indicates decreased downstream vascular resistance and orbital vasodilation, a sign of cerebral hyperperfusion.
Pulsatility Index (PI)	1.2–1.5	Reduced (Typically <1.2)	Similar to RI, reflecting lower impedance to blood flow in the cerebral circulation.
PSV Ratio (P2/P1)	Low (P2 is significantly lower than P1; ratio typically < 0.6)	Elevated (P2 approaches or exceeds P1; ratio often > 0.65–0.75, depending on gestational age)	The elevation of the second peak (P2) signifies a loss of normal vascular tone and autoregulation, leading to a “high-flow” state.
Second Peak Systolic Velocity (PSV2 or P2)	Lower absolute velocity	Elevated (A cut-off of ≥43.75 cm/s has been identified as highly discriminatory)	A direct measure of increased systolic flow velocity, correlating with increased cerebral perfusion pressure.

Note: The reference values and cut-offs presented in this table are intended as preliminary and context-dependent indicators rather than broadly generalizable standards; they should be interpreted within the specific clinical framework and gestational maturity of each case to ensure safe clinical application.

## Data Availability

Data sharing is not applicable to this article as no new data were created or analyzed in this study.

## Abbreviations

The following abbreviations are used in this manuscript:

PE	Preeclampsia
OA	Ophthalmic artery
FPR	False-positive rate
MAP	Mean arterial blood pressure
PI	Pulsatility index
RI	Resistance index
PR	peak ratio (first end-diastolic velocity to first systolic peak velocity)
PRF	Pulse repetition frequency
PlGF	Placental growth factor
PSV	Peak systolic velocity
PMDV	Peak mesodiastolic velocity
sFlt	Soluble FMS-like Tyrosine Kinase-1
UtA-PI	uterine artery pulsatility index
